# Delayed diagnosis of disseminated *Mycobacterium intracellulare* subsp. *chimaera* infective endocarditis via cell-free metagenomic next-generation sequencing: a case report

**DOI:** 10.1128/asmcr.00003-25

**Published:** 2025-05-30

**Authors:** Joseph Benigno Ladines-Lim, Wei-Teng Yang, Pablo Tebas, Judith O'Donnell, Helen Koenig, Edward Kreider, Kelly Dyer, Margaret Anwar, Emmanuel Rodriguez, Sonal Patel, Kyle Rodino, Laurel Glaser, Aaron Richterman

**Affiliations:** 1 Division of Infectious Diseases, Penn Medicine, University of Pennsylvania 215865 , Philadelphia, Pennsylvania, USA; 2 West Virginia School of Osteopathic Medicine 4518, Lewisburg, West Virginia, USA; 3 Department of Pharmacy, Penn Medicine, University of Pennsylvania 6572, Philadelphia, Pennsylvania, USA; 4 Department of Pathology and Laboratory Medicine, Perelman School of Medicine, University of Pennsylvania 192716 , Philadelphia, Pennsylvania, USA; Pattern Bioscience, Austin, Texas, USA

**Keywords:** metagenomics, *M. chimaera*, cfmNGS

## Abstract

**Background:**

*Mycobacterium intracellulare* subsp. *chimaera* infective endocarditis associated with contaminated heater-cooler units has been well documented, leading to the discontinuation of these devices in most hospitals by 2018. The rarity of this infection and its nonspecific symptoms often result in delayed diagnosis.

**Case Summary:**

We describe a 56-year-old female diagnosed with *M. intracellulare* subsp. *chimaera* infective endocarditis with disseminated intracranial abscess 7 years after aortic and mitral valve replacement. Diagnosis was achieved using cell-free microbial DNA next-generation sequencing (cfmNGS). She underwent left temporal craniotomy for abscess drainage and aortic and mitral valve replacement. Diagnosis was confirmed via mycobacterial culture from blood, brain tissue, and explanted valve tissue. Treatment included rifabutin, ethambutol, azithromycin, and amikacin, alongside a prednisone taper prescribed for a previously diagnosed undifferentiated inflammatory process. Amikacin was discontinued 6 weeks after valve surgery because of unilateral hearing loss. She remained clinically stable 5 months after valve surgery.

**Conclusion:**

This case highlights that *M. intracellulare* subsp. *chimaera* infections may continue to emerge years after heater-cool unit discontinuation, suggesting that the time window for case incidence may still be active. cfmNGS may serve as a valuable diagnostic tool for disseminated *M. intracellulare* subsp. *chimaera*. Finally, we discuss pharmacotherapeutic factors, including the need for multiple agents over long durations, in this case with specific considerations given to the dissemination of infection into the central nervous system and potential drug-drug interactions, including steroids.

## INTRODUCTION

Outbreaks of *Mycobacterium intracellulare* subsp. *chimaera* infective endocarditis associated with contaminated heater-cooler units in cardiac surgery have been well documented (1, 2). The bacterium can be aerosolized from the device’s water tanks, leading to infections with latency periods ranging from months to years. Its rarity and nonspecific symptoms often result in delayed diagnosis and misdiagnosis as alternative etiologies associated with noncaseating granulomas (1).

Although most cardiac surgical centers discontinued the implicated Sorin Stockert 3T heater-cooler unit by 2018, the device remains available with updated safety guidelines. We present a case of disseminated *M. intracellulare* subsp. *chimaera* infective endocarditis diagnosed several years after cardiac surgery identified through cell-free microbial DNA next-generation sequencing (cfmNGS).

## CASE PRESENTATION

A 56-year-old female with a history of ischemic stroke (2016), rheumatic heart disease status post aortic and mitral valve replacement (2017), and coronary artery disease status post percutaneous coronary intervention (2022) presented with altered mental status, fever, and weight loss after a 4-year history of intermittent fevers. Born in the Philippines, she immigrated to Southern Europe in the early 1990s and later to the United States in the early 2010s. Her background included employment in dining services and housekeeping until disability status in 2022. Hobbies included gardening and hiking.

In 2020, she developed whitish fingertips, dry mouth, and a lower extremity rash. Testing revealed positive anti-nuclear (>1:1,280 centromere pattern; reference range <1:80) and cyclic citrullinated peptide antibodies (114 units; reference range <20 units), leading to a diagnosis of limited scleroderma with Raynaud’s phenomenon. She later developed arthralgias and morning stiffness, initially treated with methotrexate but transitioned to tocilizumab due to elevated liver function tests. To our knowledge, no infectious testing was performed at this time.

By spring 2023, she experienced fevers, chills, and night sweats. Tocilizumab was discontinued, but symptoms continued. Abdominal ultrasound suggested chronic cholecystitis, and she developed anemia and thrombocytopenia. A transesophageal echocardiogram (TEE) identified a 1.2 cm echodensity at the aorto-mitral curtain, initially deemed insignificant. She was empirically prescribed atovaquone and azithromycin for presumed babesiosis, leading to temporary defervescence. Bone marrow biopsy revealed non-caseating granulomas, leading to the diagnosis of sarcoidosis and initiation of infliximab.

Later in 2023, worsening proteinuria and renal dysfunction prompted discontinuation of infliximab. Renal biopsy revealed membranous nephropathy with interstitial granulomatous nephritis. She was subsequently hospitalized for disseminated intravascular coagulopathy, pancytopenia, and acute suspected gallstone pancreatitis, for which she underwent a cholecystectomy and was discharged on a tapering course of prednisone.

She was re-hospitalized in early 2024 for abdominal pain and elevated liver function tests, raising suspicion for cholangitis and recurrent pancreatitis. She was empirically treated with piperacillin-tazobactam, as well as intravenous immunoglobulin and dexamethasone for her pancytopenia, leading to partial improvement. She was discharged on prednisone for presumed immune-mediated cytopenias and trimethoprim-sulfamethoxazole for *Pneumocystis jirovecii* pneumonia prophylaxis.

By fall 2024, she was evaluated by an Infectious Diseases specialist for the first time at our institution for persistent symptoms, including hearing difficulty, neck and jaw discomfort, lethargy, mild confusion, and a 25-pound unintentional weight loss over the past year. Extensive infectious disease testing was performed, including mycobacterial cultures of induced sputum, interferon gamma release assay, rapid plasma reagin, serologies for *Bartonella quintana*, *Brucella*, and *Coxiella burnetii,* tests for *Schistosoma*, *Strongyloides*, *Blastomyces*, *Coccidioides*, as well as *Histoplasma* and *Aspergillus* antigens, β-D-glucan, stool ova and parasites, and cfmNGS from plasma via the Karius Test, which typically reports results within 2 days (3).

**Fig 1:**
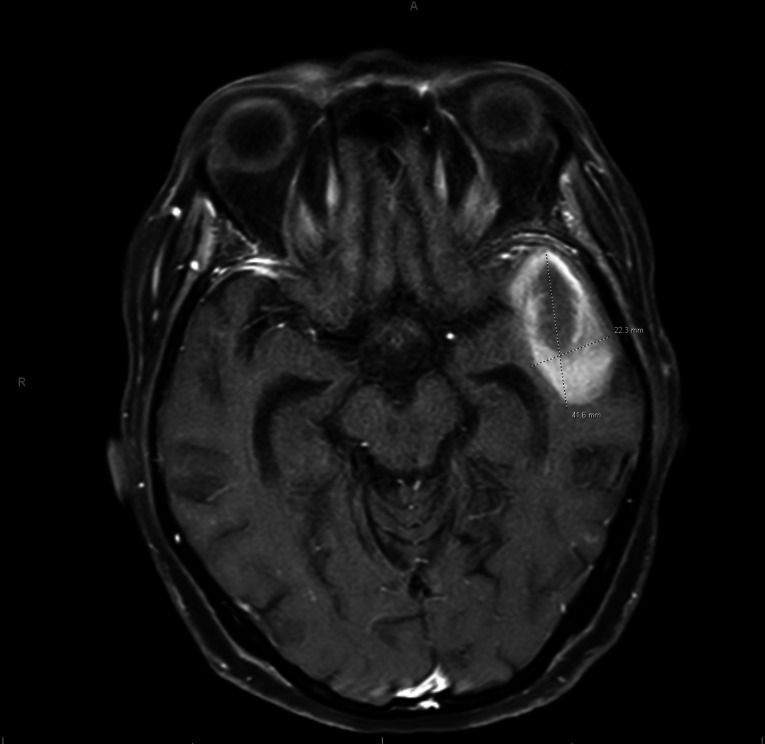
Magnetic resonance imaging of the head with and without IV contrast, showing complex cystic/proteinaceous structure without clear evidence of enhancing component or surrounding edema or mass effect, measuring about 4.2 × 2.2 cm in size, consistent with evolving subacute to chronic hematoma, neoplasm, or abscess with atypical features.

A few weeks later, she was admitted to our hospital for altered mental status, lethargy, and persistent fevers. A non-contrast computed tomography scan of her head revealed left temporal lobe edema interpreted as a mass or recent infarct. Magnetic resonance imaging demonstrated a complex hemorrhagic, proteinaceous structure without clear surrounding edema or enhancement, suggestive of an evolving subacute to chronic hematoma, neoplasm, or abscess (Fig. 1). She was started on empiric vancomycin, with 1 g loading dose and 500 mg every 12 h thereafter guided by area under the curve calculations and target trough level of 15–20 mcg/mL, and cefepime 2 g every 12 h (adjusted from every 8 h given her renal function at the time).

Neurosurgery and Infectious Diseases were consulted. During this hospitalization, her outpatient cfmNGS was noted to be positive for *M. intracellulare* subsp. *chimaera* (3,788 molecules/microliter), prompting reassessment of her clinical history; of note, this pathogen was not specifically on the differential at the time that testing was sent. Given her 2017 valve surgery (presumably using a Sorin heater-cooler unit, though not confirmed given this was done at an outside medical center) and prior TEE findings, there was strong suspicion for *M. intracellulare* subsp. *chimaera* infective endocarditis with dissemination to the central nervous system.

Antibiotics were transitioned to rifabutin 300 mg daily (selected to minimize interactions with prednisone), ethambutol 800 mg daily, azithromycin 500 mg daily, and amikacin 15 mg/kg daily for a tentative 18-month course. A lumbar puncture revealed cerebrospinal fluid studies showing protein 100 mg/dL and glucose 31 mg/dL, though cerebrospinal fluid cultures remained negative. Given the concern for an intracranial infectious process, she underwent a left temporal craniotomy for biopsy and drainage. Cultures from the craniotomy biopsy eventually grew an *M. avium-intracellulare* (MAI) complex after nearly 2 weeks with susceptibility testing performed, despite an initially negative acid-fast bacilli fluorochrome stain presumed to represent *M. intracellulare* subsp. *chimaera* as part of the MAI complex given her cfmNGS testing positive for *M. intracellulare* subsp. *chimaera*. Mycobacteria blood cultures eventually also became positive for MAI complex (positive gram stain at nearly 2 weeks and final culture results at nearly 6 weeks), supporting the diagnosis of disseminated infection.

**Fig 2:**
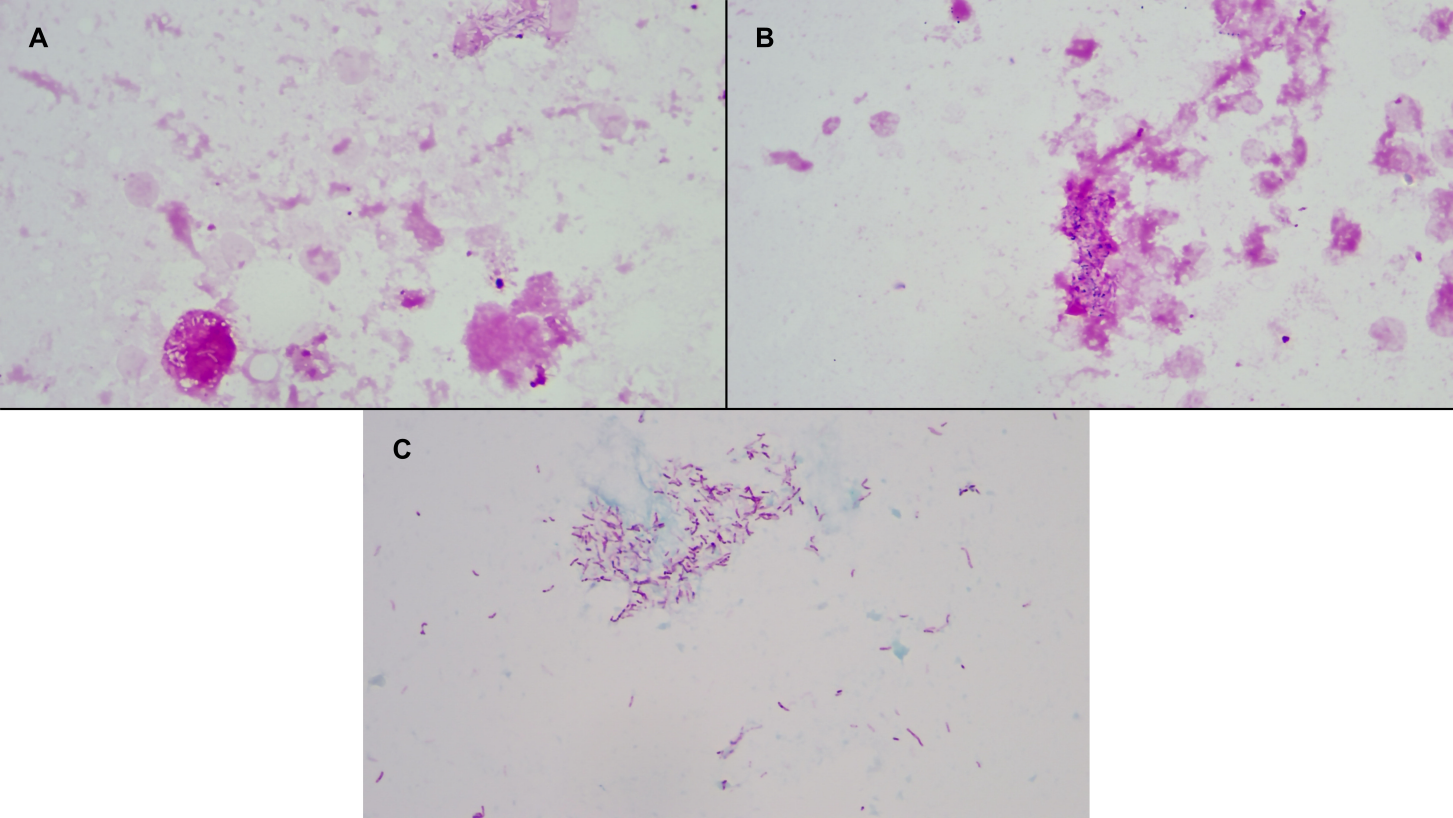
Gram (A and B) and Kinyoun (C) stains of explanted aortic valve showing many acid-fast bacilli, consistent with infective endocarditis secondary to *Mycobacterium intracellulare* subsp. *chimaera*.

Cardiac Surgery was consulted, and she subsequently underwent aortic and mitral valve replacements. Cultures from the explanted prosthetic aortic and mitral valves grew MAI complex also presumed to be *M. intracellulare* subsp. *chimaera* given her cfmNGS testing (Fig. 2). Postoperatively, she developed lactic acidosis, prompting emergency laparotomy, though no definitive source was identified. Her post-operative course was further complicated by respiratory failure requiring mechanical ventilation, multifocal pneumonia, loculated pleural effusion requiring chest tube placement, and atrial fibrillation. She was eventually extubated and gradually improved. After hospitalization for 47 days, including 24 days after valve surgery, she was discharged to a skilled nursing facility for rehabilitation on antibiotics and a prednisone taper.

At the 16-day follow-up, she reported left ear hearing loss and tinnitus, prompting discontinuation of amikacin due to ototoxicity; she remained on rifabutin 300 mg daily, ethambutol 800 mg daily, and azithromycin 500 mg daily. Currently, she remains stable 5 months after valve surgery, with baseline mental status and weight restored.

## DISCUSSION

This case illustrates the profound diagnostic challenges posed by *M. intracellulare* subsp. *chimaera* infections, particularly in patients with complex medical histories. Initially, the patient’s noncaseating granulomas on bone marrow biopsy led clinicians to suspect sarcoidosis—a diagnosis further reinforced by her rheumatologic markers and clinical features, such as arthralgias and elevated liver enzymes. The identification of a 1.2 cm echodensity on TEE more than a year before the final diagnosis raised the possibility of an occult infection that went unrecognized. In hindsight, the entire disease course could be attributed to the final diagnosis of *M. intracellulare* subsp. *chimaera* infection, particularly given the timing of her cardiac surgical history, which may have been diagnosed earlier if traditional culture-based testing had been obtained. Of note, while noncaseating granulomas are a known histopathologic feature of this infection, additional testing was not pursued that also could have identified the infectious process, including acid-fast stain or polymerase chain reaction and sequencing on the surgical pathology sample, or mycobacterial cultures from bone marrow or blood. All could have plausibly yielded the diagnosis, as has been documented previously for mycobacteria species (4).

However, without the abovementioned confirmatory testing, it likely would have been challenging to start empiric treatment in this case given the unpredictability of the organism’s antibiotic susceptibilities and the innate toxicities of oft-used therapies, among other reasons, which we describe later in this section. Ultimately, plasma cfmNGS provided the pivotal clue by rapidly identifying *M. intracellulare* subsp. *chimaera*, underscoring the critical role of advanced molecular diagnostics in detecting elusive pathogens that are notoriously slow to culture. Still, from the perspective of diagnostic stewardship, the optimal timing for obtaining plasma cfmNGS or other next-generation sequencing testing in relation to other diagnostic tests, such as more traditional cultures, is not known and may vary with clinical context and the underlying true infectious pathogen; this is certainly a topic that warrants further investigation. We note that at our center, all plasma cfmNGS tests are sent after discussion with an Infectious Diseases specialist and/or the clinical microbiology laboratory directors; we acknowledge that this approval process may vary at other institutions.

The patient’s case also illustrates the significant morbidity associated with *M. intracellulare* subsp. *chimaera* endocarditis, including multiorgan involvement, central nervous system lesions, and systemic symptoms that mimic autoimmune and inflammatory disorders. The presence of *M. intracellulare* subsp. *chimaera* in the explanted prosthetic valves and positive blood cultures confirmed disseminated disease. Given the slow-growing nature of non-tuberculous mycobacteria and the difficulty in culturing these organisms, a high index of suspicion is essential in patients with prior cardiac surgery and unexplained systemic illness.

Treatment of *M. intracellulare* subsp. *chimaera* infections is inherently challenging due to the pathogen’s intrinsic resistance to many conventional antibiotics. A prolonged course of multidrug therapy, often exceeding 12 months, is typically required. Standard regimens include macrolides (azithromycin or clarithromycin), rifamycins (rifabutin or rifampin), ethambutol, and an aminoglycoside (amikacin) (1, 2), although in our patient, amikacin was discontinued due to concerns for ototoxicity. Outcomes generally remain guarded, particularly in cases of prosthetic valve endocarditis requiring surgical intervention, with rates of relapsed infection estimated at 30–50% and mortality estimates ranging from 20 to 67% (1).

In our patient’s case, the initial clinical improvement following empiric treatment with atovaquone and azithromycin likely contributed to a delay in considering an alternative diagnosis. Moreover, her rheumatologic profile and laboratory findings obscured the clinical picture, as disseminated *M. intracellular* subsp. *chimaera* infection can present with hepatic involvement, nephritis, cytopenias, neurologic complications, and granulomas—features that overlap with those seen in sarcoidosis and other inflammatory disorders (1).

To our knowledge, cfmNGS has been used to diagnose only one other case of *M. intracellulare* subsp. *chimaera* infective endocarditis 2 years after cardiopulmonary bypass surgery (5). Metagenomic next-generation sequencing of blood—similar to cfmNGS of plasma but distinct in that it uses an untargeted sequencing approach to analyze all genetic material, including RNA (6)—has also been reported in *M. kansasii* endocarditis,^7^ *M. avium* pneumonia (8), and disseminated infections with *M. wolinskyi*, *M. goodii*, *M. smegmatis*, and *M. mageritense* (8). Karius has also published their own experience, identifying 18 *M*. *intracellulare* subsp. *chimaera* cases, although specific contexts were not provided (9). Our case adds to a growing body of evidence supporting the utility of advanced sequencing technology in diagnosing otherwise confounding cases. Importantly, the relatively rapid turnaround time for cfmNGS performed via the Karius Test, typically within 2 days, compared to up to 6 weeks required for culturing slow-growing mycobacteria, such as *M. intracellulare* subsp. *chimaera* or 1 to 3 weeks for broad-range polymerase chain reaction testing, as well as any additional time needed for procedural sampling, facilitated prompt initiation of therapy during hospitalization.

Optimal treatment regimens and duration for *M. intracellulare* subsp. *chimaera* infections remain unclear (1, 10). Although adjunctive corticosteroids are often used for immune reconstitution inflammatory syndrome in disseminated cases, there are no standardized guidelines for dosing, and the potential for drug-drug interactions must be carefully considered, such as the use of rifamycin to minimize interaction with prednisone in our patient’s case. Central nervous system involvement further complicates treatment, as ensuring adequate antimicrobial penetration into brain tissue is an ongoing challenge.

### Conclusion

This case highlights the importance of considering *M. intracellulare* subsp. *chimaera* infection in patients with prior cardiac surgery, unexplained systemic symptoms, and prosthetic valve involvement. As more patients present with delayed manifestations of infection, ongoing vigilance and early application of advanced molecular diagnostics will be critical in improving recognition and outcomes. cfmNGS was critical for diagnosis in our case, adding to the limited body of evidence supporting the use of advanced sequencing technology in diagnosing mycobacterial infections notably in accelerated fashion compared with traditionally used cultures.
